# Large animal models of cardiovascular disease

**DOI:** 10.1002/cbf.3173

**Published:** 2016-02-24

**Authors:** H. G. Tsang, N. A. Rashdan, C. B. A. Whitelaw, B. M. Corcoran, K. M. Summers, V. E. MacRae

**Affiliations:** ^1^ The Roslin Institute, Royal (Dick) School of Veterinary StudiesThe University of Edinburgh, Easter BushMidlothianSCTUK; ^2^ Royal (Dick) School of Veterinary StudiesThe University of Edinburgh, Easter BushMidlothianSCTUK

**Keywords:** cardiovascular disease, large animal models, calcific aortic valve disease, aortic stenosis, vascular calcification, Marfan syndrome, genetic engineering

## Abstract

The human cardiovascular system is a complex arrangement of specialized structures with distinct functions. The molecular landscape, including the genome, transcriptome and proteome, is pivotal to the biological complexity of both normal and abnormal mammalian processes. Despite our advancing knowledge and understanding of cardiovascular disease (CVD) through the principal use of rodent models, this continues to be an increasing issue in today's world. For instance, as the ageing population increases, so does the incidence of heart valve dysfunction. This may be because of changes in molecular composition and structure of the extracellular matrix, or from the pathological process of vascular calcification in which bone‐formation related factors cause ectopic mineralization. However, significant differences between mice and men exist in terms of cardiovascular anatomy, physiology and pathology. In contrast, large animal models can show considerably greater similarity to humans. Furthermore, precise and efficient genome editing techniques enable the generation of tailored models for translational research. These novel systems provide a huge potential for large animal models to investigate the regulatory factors and molecular pathways that contribute to CVD *in vivo*. In turn, this will help bridge the gap between basic science and clinical applications by facilitating the refinement of therapies for cardiovascular disease. 2016 The Authors. Published by John Wiley & Sons Ltd.

## Introduction

The mammalian cardiovascular system is a vast network of specialized structures and vessels, which allows blood and other important molecules to be transported throughout the body. Central to the system is the four‐chambered heart that acts as a muscular pump to permit the movement of blood. Four cardiac valves ensure that blood flows through the heart and into the arteries in only one direction 1. The veins and arteries comprise three concentric tubes: an outer connective tissue layer (tunica externa), a middle smooth muscle cell layer (tunica media), which is thinner in the veins than the arteries and an inner endothelial cell layer (tunica intima) 2.

Cardiovascular disease (CVD) is a leading global cause of morbidity and mortality 3. The American Heart Association (AHA) reported that CVD accounted for approximately one in three deaths in the United States in 2011 4. Additionally, 34% of CVD‐attributed deaths occurred before 75 years of age, which was below the present average life expectancy of 78.7 years 4. According to the World Health Organization (WHO), some of the top cardiovascular‐related causes of premature death include coronary heart disease, chronic obstructive pulmonary disease, and stroke 5.

In recent decades, both invasive and non‐invasive therapies of CVD have advanced considerably. This advancement has been facilitated by basic research, and progressed with clinical studies 6. Nowadays, it is becoming more apparent that understanding biological systems at the basic scientific level is important in order to provide clinicians with new approaches and tools for the assessment and treatment of their patients 6.

Within the field of cardiovascular research, new interventional strategies range from experimental procedures for testing new implantations and devices, to more specific studies of underlying mechanisms of particular CVDs. In the development of these strategies and basic research, the role of animal models of CVD is especially important. This review aims to look at some of the cardiovascular issues in today's world, such as heart valve disease and vascular calcification, the expanding research resources made available through the use of large animal models and the potential of novel genetic engineering technologies in this field.

## Animal Models of CVDs

Animal models are important for discerning the aetiology and pathogenesis of human diseases with the purpose of developing novel disease preventions and therapies 7. Many of our achievements on the treatment and management of CVDs have been made through the use of experimental animal models. These disease models have helped in outlining the pathogenesis, progression and mechanisms behind CVDs at the molecular and cellular levels, enabling the development of many effective treatment approaches.

A number of models exist to address cardiovascular complications, such as atherosclerosis and other cardiac diseases, where similar pathologies have been recreated in different species, including large and small animals. Although no model can entirely replicate the complexities seen in human pathologies, they are crucial in assessing mechanisms of disease, as well as evaluating novel diagnostic technologies, preventions and therapies 7, 8. Model organisms are used predominantly to improve human health, and to enable translatable scientific discoveries with practical applications. Large animals can facilitate in these goals, as they exhibit disease characteristics similar to humans, giving mechanistic insight into the biological and pathological processes. Additionally, they enable us to obtain direct information about specific physiological events, and studies of diseases with respect to a control group are possible in order to observe the effects of particular variables, treatments, and modified factors. This is in contrast to human studies, in which appropriate age and sex matched control sample groups are often very difficult to obtain.

The current use of small rodents as the main model of human diseases is widespread, and they are a popular choice of species for several reasons. They are relatively cost effective and easy to maintain, and can provide large litters; thus, studies can be given adequate power by the use of appropriate numbers of animals. The ability to genetically modify mice through either the ablation or overexpression of a gene of interest has made them indispensable in teasing apart the mechanisms underpinning CVD.

Nonetheless, limitations do exist, and significant cardiovascular differences are apparent between humans and rodents 9. In light of the important role of inflammation in several CVDs, differences between species should be considered. One of the most drastic disparities between humans and mice is the response to bacterial lipopolysaccharides, which is five orders of magnitude higher in humans 10. This contrasting response has been attributed to the differential composition of mouse serum 11: in mouse blood the predominant population of leukocytes is lymphocytes, whereas human blood is neutrophil rich 12. Interestingly, pigs show greater similarity to humans in this regard, whereby neutrophils are also the predominant circulating blood cell population 13. Furthermore, during delayed‐type hypersensitivity testing neutrophils surround the antigen followed by an influx of mononuclear cells resulting in a predominantly macrophage and T‐cell lesion in humans 14. However, in mice a comparable test results in a predominantly neutrophil lesion 15. The small size of rodents makes them easy to handle and offers key advantages such as the application of intra vital microscopy in *in vivo* inflammatory studies 16. Additional imaging techniques are frequently complicated by the smaller size, and smaller volumes of circulating blood also make repeat sampling challenging in these studies.

Although employing large animals may involve higher costs, because of their size and husbandry needs when compared to smaller models, their importance in the field of human diseases is evident as they have more cardiovascular similarities in terms of anatomy, physiology and size to humans than the rodent species. The ability to apply human‐like settings to model animals increases the chances of bench findings translating to effective treatments. This includes using human clinical equipment and surgical techniques. For example pigs have been used for decades to develop surgical procedures for implementation in humans, and pig valves are used in some cases of human valve failure 17. In addition, their larger size provides a better choice for imaging and tissue engineering studies. Studies utilising large animal models can illuminate the biological pathways and mechanisms to facilitate the refinement of CVD therapies. Despite these advantages, there are significant challenges to the use of large animal models in addition to costs. These include the availability of antibodies and assays specific to these species. However, with increasing use of large animals the increased demand should bring about an increase in availability of these products. Compared to the mouse there are few transgenic large animals. However, new gene editing technologies allow the establishment of precise and efficient gene editing techniques that, as described later in this review, should enable the generation of tailored large animal models of human disease.

## The Cardiovascular System, Diseases and Insights

### Valvular heart disease (VHD)

Valvular heart disease (VHD) encompasses a range of cardiovascular conditions, accounting for 10–20% of all cardiac surgeries in the United States 18. As the ageing population continues to increase, so does the prevalence of patients with degenerative valve disorders 4. In addition, the morbidity and mortality rates of open‐heart surgery, the main approach taken for patients with VHD, can be high, providing challenges to reconstructive procedures 6. Better understanding of the function of the valves and the perturbations that lead to disease is imperative to the future provision of surgical and therapeutic interventions.

**Figure 1:**
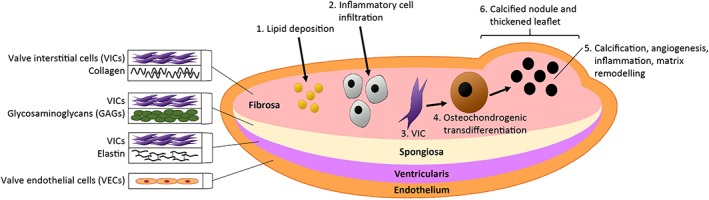
Simplified cross section of the aortic valve showing progression of aortic valve calcification. Valve endothelial cells (VECs) line the valve leaflet surface. The inner layers of the valve consist of the fibrosa, spongiosa and ventricularis. The principal cell type within each layer is the valve interstitial cells (VICs). The fibrosa contains collagen (Types I and III), the spongiosa contains glycosaminoglycans (GAGs) and the ventricularis—elastin fibres. In calcific aortic valve disease (CAVD), often the fibrosa layer becomes calcified and thickened. This may be because of lipid deposition and inflammatory processes which trigger the osteochondrogenic transdifferentiation of VICs. Calcium deposition then occurs, forming bone‐like material as neovascularization around the calcified lesions and remodelling of the extracellular matrix occurs. This results in the formation of calcified nodules and the thickening of the valve leaflet

There are four cardiac valves: the mitral (bicuspid) valve and aortic semilunar valve on the left side of the heart, and the tricuspid valve and pulmonary semilunar valve on the right side. The heart valve leaflet structure consists of cellular and extracellular components. Extracellular components include collagen, glycosaminoglycans (GAGs) and elastin present in the three layers of the valve: the fibrosa, spongiosa and ventricularis, respectively (Figure 1) 19, 20, 21, 22. Valve surface endothelial cells (VECs) and the inner valve interstitial cells (VICs) are the principal cell types found in the cardiac valves 19, 20, 23.

Over 60% of valve disease mortality is because of semilunar valve dysfunction, especially of the aortic valve, with approximately 50 000 valve replacement or repair procedures reported each year in the USA 24. Semilunar valve diseases affect all ages, from congenital valve defects in neonates and children, to the increasing number of elderly with calcified valves 24. These dysfunctional heart valves most often require surgical replacement using mechanical or bioprosthetic valves which are prone to failure over time from structural or thrombosis‐related problems 24.

Although many studies into valvular biology use adult aortic valve tissues and cells from either humans or animals, it is clear that the subject's age is important in order to assess age‐specific pathologies and conditions 24. An earlier study reported numerous age‐related changes in the extracellular matrix (ECM) composition and mechanical properties of the aortic valves, in addition to valve cell phenotypes 24. The ECM is the cell‐synthesized structural backbone of connective tissue. It provides a structural frame for mesenchyme‐derived tissues, in addition to regulating interactions between numerous growth factors and cell surface receptors 25, 26. Within the ECM are elastic fibres, collagen fibrils and microfibrils, which contain components including elastin, collagen and the fibrillins 27. In the cardiovascular system, this complex meshwork also ensures normal cardiovascular function by providing the biomechanical characteristics of the blood vessel walls 27. Substantial tissue growth and remodelling occur before adulthood 24. In foetal development, trilaminate ECM structures or highly aligned elastin and collagen that are evident in adult valves are not yet present in the aortic valves 24. The ECM is also an essential component in the cardiac valves, where its disruption has been reported in valve diseases, as will be mentioned later on. As valvular diseases continue to increase in the elderly population, improving our understanding of how valve cells and the ECM functions change throughout ageing is crucial, as well as distinguishing their responses to surrounding environments in various physiological states, such as those pertaining to vascular calcification.

The function of the cardiac valves is of crucial importance. However, the aortic valve is especially vital as it is a partition between the left ventricle and the aorta, at the level where the coronary arteries arise. Therefore, significant clinical complications occur when congenital defects and chronic disorders relating to the aortic valve are present, as current diagnostic and therapeutic strategies are inadequate. Although there have been major advancements in the last decade or so in valvular biology, there are still many pieces to be found to form a clear picture of the pathological mechanisms that impair valvular function.

### Models of calcific aortic valve disease (CAVD)

Calcific aortic valve disease (CAVD) is a progressive disorder involving valve leaflet thickening (aortic sclerosis), leading to severe calcification with impaired leaflet motion (aortic stenosis) 28. Aortic stenosis is a major form of CVD, along with hypertension and coronary artery disease, in the Western world 18, 29. Almost 30% of adults above 65 years have aortic stenosis, and approximately 50% in those above 85 years 30, 31. It develops from progressive leaflet calcification, causing gradual restriction of the opening of the leaflets 18. Aortic stenosis shares similarities with atherosclerosis, for example, their risk factors include age, diabetes, hypertension, obesity, increased low‐density lipoprotein (LDL) cholesterol and lipoprotein(a), as well as smoking 18, 31, 32, 33, 34. Pathological studies of human aortic stenosis have identified valvular lesions containing inflammatory cells and calcific deposits similar to those found in atherosclerotic plaques 33. Once symptoms develop as a result of increasing stenosis severity, including angina and heart failure, there is a higher risk of sudden death with the average survival of only 2–3 years 18, 35.

**Figure 2:**
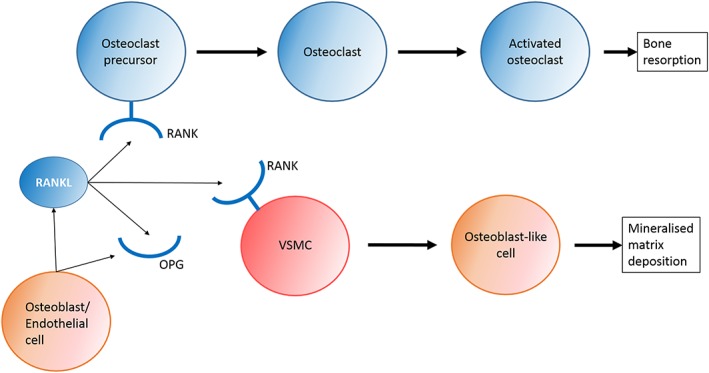
Simplified diagram showing potential RANK/RANKL/OPG involvement in bone remodelling and in vascular calcification. Receptor activator of nuclear factor kappa‐B ligand (RANKL) from osteoblasts or endothelial cells binds to the Receptor Activator of Nuclear Factor kappa‐B (RANK) of osteoclast precursors, or vascular smooth muscle cells (VSMCs). This leads to differentiation into mature osteoclasts in the bone, which are involved in bone resorption, whereas in vascular calcification, VSMCs undergo a phenotypic transition into osteochondrogenic cells that can deposit mineralized matrix. Osteoprotegerin (OPG) is the decoy receptor for RANKL, and a potential inhibitor for mineralization

**Figure 3:**
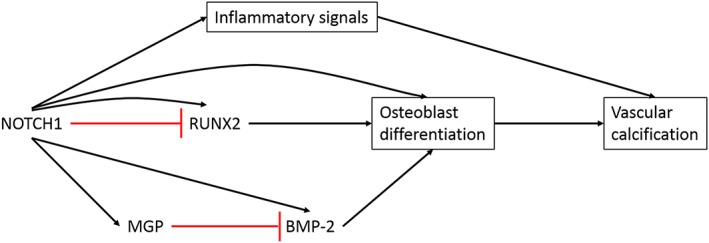
Simplified diagram showing potential NOTCH1 involvement in vascular calcification. NOTCH1 signalling may be involved in the inhibition, as well as the promotion, of vascular calcification through additional factors. Black arrows indicate stimulatory effects, whilst red lines indicate inhibitory effects

Studies assessing the biological and structural changes in aortic valves have predominantly used mouse models. Techniques used have included staining for calcium deposition, quantitative real‐time PCR (qRT‐PCR) to examine changes in mRNA levels for specific genes, protein quantification and enzymatic activity 36. To date, there are reports of pro‐osteogenic signalling cascades thought to contribute to the initiation and progression of aortic stenosis. Signalling molecules include bone morphogenetic proteins (BMPs), Wnt/β‐catenin and transforming growth factor‐β (TGF‐β) although the role of TGF‐β in osteogenic signalling is not clear 36. The RANK/RANKL/OPG pathway is also thought to be involved in the calcification process, which involves complex interactions between receptor activator of nuclear factor kappa B (RANK), RANK ligand (RANKL), and osteoprotegerin (OPG) (Figure 2) 36, 37. Matrix remodelling may also be involved in the expansion of calcified plaques and pro‐inflammatory processes, through alterations in matrix metalloproteinases (MMPs) and elastin fragments produced by cathepsins 36. Furthermore, the NOTCH1 pathway has been implicated as a regulator of valve calcification, through the repression of the osteoblast transcription factor Runt‐related transcription factor 2 (RUNX2) (Figure 3) 38. This suggests an inhibitory role of NOTCH1 in valvular calcification. Additionally, a number of ECM proteins have been found to have roles in CAVD including collagen, elastin and GAGs, where changes in their expression have impacts on cellular processes, and also cause valve leaflet thickening 39.

Pro‐mineralization processes can also be regulated by circulating calcification inhibitors, including matrix γ‐carboxyglutamic acid or matrix Gla protein (MGP), which inhibits BMP signalling 40. CAVD patients were also found to have less circulating MGP 41. Another circulating inhibitor of calcification, Fetuin‐A, binds clusters of calcium and phosphate, preventing their uptake into cells 42. Circulating levels of Fetuin‐A have been shown to be reduced in peritoneal dialysis patients displaying rapid development of valvular calcification 43. Furthermore a cross‐sectional study of 970 patients with coronary artery disease found an inverse relationship between circulating Fetuin‐A levels and mitral and aortic valve calcification 44. Aortic stenosis in patients with CAVD has also been associated with increased plasma levels of potential promoters of calcification, such as the non‐collagenous bone matrix protein osteopontin (OPN), a pro‐inflammatory glycoprotein that regulates calcium deposition by osteoblasts 45.

The mechanism for CAVD development is similar to skeletal bone formation (ossification). CAVD progression is regulated by cells within the valve that develop an osteoblast‐like phenotype 46. Within calcified aortic valves, markers of bone differentiation have also been discovered, including RUNX2, OPN, osteocalcin (OCN) and bone sialoprotein 46, 47. RUNX2 expression is a marker for osteoblast differentiation, whilst OCN is a late marker of calcification in osteoblastogenesis 48, 49. Intriguingly, ossification, the active process of bone tissue repair and remodelling, is observed in end‐stage VHD 50. In addition to the presence of mature lamellar bone and infiltration of inflammatory cells, BMPs ‐2 and ‐4 have been found in calcified valves 50. BMP‐2 is a potent osteogenic differentiation marker, a member of the TGF‐β superfamily, and has been found to be involved in calcification following the repression of NOTCH1 signalling in sheep aortic VICs 51. Mutations in *NOTCH1* have been linked with the presence of aortic valve calcification in human and mouse studies, although the disease was much milder in the latter 51. A recent study of NOTCH1 in human aortic VECs found that this factor positively regulated MGP 52. Shear stress was simulated by media flow conditions *in vitro* and was found to activate *NOTCH1* expression 52. Because MGP can inhibit BMPs, this may be the route through which NOTCH1 represses BMP‐2. Intriguingly, it has been reported that NOTCH1 induces the differentiation and calcification of vascular smooth muscle cells (VSMCs) through a BMP‐2 driven mechanism (Figure 3) 51. However, the role of NOTCH1 in the calcification of valves and vessels remains to be fully understood, and requires further investigation.

The mechanisms underpinning the process of valvular calcification have yet to be fully elucidated. However, an ‘initiation’ and ‘propagation’ phase has been described 53. It may be that in response to valvular damage, such as through endothelial disruption via shear stress, lipid deposition and inflammation occur, which subsequently triggers calcification (Figure 1) 54, 55, 56. Neovascularization was also found in calcified valves, suggesting that this is important in CAVD 50, with new vessels commonly found in areas of inflammation around calcified deposits 57. Haemorrhage has been associated with neoangiogenesis, macrophage infiltration and accelerated disease progression in patients with advanced aortic stenosis 58. Nevertheless, the roles of the factors and pathways involved in VHDs and other CVDs, including those briefly mentioned above, remain unclear. More information into the early stages of disease initiation and progression is required to understand the mechanisms of CAVD.

Currently, the only option to treat CAVD is through valve replacement surgery. This reflects our lack of understanding of the early pathobiological processes, even though later stages of the disease are well described. Additionally, as the implicated regulators of ectopic calcification are required for normal bone mineralization, establishing a therapeutic strategy that does not affect bone is crucial. And so, uncovering the factors and mechanisms that are important during the initiation and progression of these diseases facilitated by animal models is required for developing such treatments and interventions. Many studies into aortic stenosis utilize the mouse as a model 36. However, the size of the aortic valves limits the amount of material available for examining the molecular mechanisms behind CAVD. Although diseased human valve samples can be obtained relatively easily, acquiring healthy samples is nearly impossible. In contrast, healthy valve samples from animals are far more accessible, can be used as a healthy control and can provide a source of valve cells that can be isolated for experimentation 59, 60.

### Large animal models of cardiovascular dysfunction.

Models of CAVDs (Table 1) have been established in a range of animals including the pig, rabbit and dog (reviewed in 61, 62, 63, 64, 65, 66). For cardiac valve specific studies, aortic valve cells, predominantly the VECs and VICs, have been isolated and cultured from various large animals, including the pig, cow, sheep and dog 60, 62, 67, 68, 69, 70, 71, 72, 73. A rabbit model of hypertension was used to study mild aortic valve stenosis 74. In this study, echocardiography was used to assess the morphology and function of aortic valves, as well as left ventricular mass 74. This study demonstrated that hypertensive rabbits tended to show reduced aortic valve area and increased valve thickness. Although the rabbit is often used to study VHD, the atherosclerotic lesions that are formed do not simulate those found in humans, limiting its suitability as a model of CVD 66.

**Table 1:** Examples of large animal models of calcific aortic valve disease (CAVD)

Animal model	Key findings	Reference
*Pig*	Aortic side valve endothelial cells (VECs) may be more susceptible to calcification.	Sider et al. 2014 61
	High fat/high cholesterol (HF/HC) diet induced thick proteoglycan lesions in the fibrosa layer (aortic side of valve) in an up to 5‐month study.	Simmons et al. 2005 78
	HF/HC diet induced calcification in atherosclerotic lesions in an up to 48‐week study.	Gerrity et al. 2001 77
*Rabbit*	Mild aortic valve stenosis in hypertensive rabbits, increased valve thickness and inflammation nodules, hypertrophy of valve after 4 months.	Cuniberti et al. 2006 74
	High cholesterol diet for 20 and 40 weeks, atherosclerotic lesions present in aortic valves, with increased lipid deposition, inflammatory cell infiltration, osteopontin (OPN) deposition, changes in collagen and elastin distribution, and mineralization.	Cimini et al. 2005 288
*Dog*	Canine aortic valve interstitial cells (VICs) spontaneously formed apparent calcified nodules containing hydroxyapatite within 2–3 weeks.	Mohler et al. 1999 62
*Sheep*	The inhibition of bone morphogenetic protein 2 (BMP‐2) by Notch1 signalling in sheep aortic VICs is a potential mechanism by which aortic valve calcification is subdued.	Nigam and Srivastava 2009 289

The pig can develop spontaneous lesions in the vasculature and cardiac valves, and has been widely used to study atherosclerosis 75. The porcine cardiovascular system shares many similarities with that of humans, including heart anatomy and tri‐layered aortic valve leaflets, as well as similar lipid profiles and lipoprotein metabolism 76, 77. These attributes highlight the pig as an ideal model for CAVD. Currently there are a number of research groups working to characterize cellular and molecular components in porcine valves. For example, aortic and ventricular side aortic VECs from adult male pigs have been compared, using microarray and qRT‐PCR to measure gene expression 78. In this study, side‐specific expression differences were found between the aortic and ventricular VECs 78. Interestingly, higher expression was noted in the aortic side of the valve of genes associated with vascular calcification and skeletal development, such as BMP‐4 78. Lower expression of factors shown to inhibit ectopic calcification was also observed in the aortic side VECs, including OPG, C‐type natriuretic peptide (CNP) and chordin (an inhibitor of the osteoinductive activity of BMPs) 78. This may permit aortic side‐specific vulnerability to calcification. In addition to this, greater expression of antioxidative genes and an absence of differential expression of pro‐inflammatory factors on the aortic side suggests potential protection in the normal valve against lesion development and inflammation 23, 78.

A porcine model of early aortic valve sclerosis has also been assessed 61. In this investigation, pigs were fed either a standard or high fat/cholesterol (HF/HC) diet for 2–5 months. Swine fed on the HF/HC diet developed significantly thicker lesions on the aortic side of coronary aortic valve leaflets, with histologically opaque regions consisting of proteoglycans, collagen and elastin, within the fibrosa layer as similarly observed in early human CAVD 61. Increased expression of osteochondrogenic markers including SRY (sex determining region Y)‐box 9 (SOX9) and Msh Homeobox 2 (MSX2) has been observed in dense proteoglycan‐rich lesion onlays with the HF/HC diet 61 as have complicated atherosclerotic lesions with ectopic calcification 77. Furthermore, there appears to be a higher susceptibility of the aortic side of the aortic valve leaflet to calcification and disease lesions 79. Additional investigations are required to identify the side‐specific components and mechanisms that may underlie these observations in the aortic side of the leaflets.

As inflammation plays a key role in the initiation and development of valve calcification, a gene profile of porcine aortic VICs (PAVICs) under elevated pressure was generated to study expression of inflammatory genes, with the results showing similarities to those seen in CAVD 80. The ECM protein matrix metallopeptidase 3 (MMP3) and pro‐inflammatory cytokine interleukin 6 (IL‐6) were amongst those found to be upregulated in this study 80. Furthermore, the inflammatory gene network revealed was associated with the upregulation of tumour necrosis factor alpha (TNFα) 80.

The similarities of early stages of aortic stenosis to atherosclerosis through the shared processes of lipid deposition, inflammation and calcification originally led to the idea that statins may be beneficial in CAVD patients. In clinical trials in patients, however, statin therapies surprisingly failed to produce reduction in the progression of aortic stenosis, despite significant decreases in serum LDL cholesterol levels 81, 82, 83. While both lipid deposition and inflammation may be important processes in aortic stenosis, it may be likely that the accumulation and propagation of calcium crystals drives disease progression. Future therapies against aortic stenosis may involve lipid‐lowering and calcification inhibiting effects, such as through a combination of statins and mineralization inhibitors.

### Vascular calcification and models

Vascular calcification is a disease of abnormal mineral metabolism, in which calcium phosphate crystals, in the form of hydroxyapatite (HA), are deposited in blood vessels 84, 85, 86. It is a hallmark feature in ageing, hypertension and atherosclerosis 86, 87, 88. For example, coronary artery calcification (CAC) predicts atherosclerotic burden in the arteries, which can be measured by computerized tomography (CT) 4. The presence of CAC is an indicator of the presence of atherosclerotic plaque 4. The pathological process of vascular calcification is a major, independent risk factor of cardiovascular mortality 85.

Calcification can develop in the tunica media and/or the intima layers of blood vessels, resulting in the thickening and loss of elasticity of arterial walls 86, 89. Intimal calcification is typically found in large vessels and coronary arteries, and it involves intimal hyperplasia and atherosclerosis 90. For medial calcification, dense calcium sheets form in the tunica media between VSMCs, which have been found to contain bone components, including bone trabeculae and osteocytes 90. The latter form of calcification is most frequently exhibited in distal vessels of patients with advanced ageing, diabetes and kidney failure 90.

Other than the blood vessels, calcification of the vascular system can also be found in the myocardium and the cardiac valves (as described previously), and ectopic calcification in these regions is associated with clinical symptoms 84, 91. Ectopic vascular calcification impairs blood flow and blood vessel compliance, making it an independent and strong predictor of death through cardiovascular risks, such as arterial hypertension, left ventricular hypertrophy and cardiomyopathy 89, 92, 93.

Vascular calcification can be induced through the loss of mineralization inhibitors, as well as the initiation of ectopic bone formation 94. This process shares many similarities with physiological bone mineralization, where there are proteins associated with bone formation being generated by VSMCs 90, 95, 96. Extensive investigations in the last two decades have shown that pathological vascular calcification is a tightly regulated process, where vascular cells may acquire osteoblast‐like functions 86. Vascular cell calcification can be stimulated by the same group of genes as those expressed during bone formation 97. Atherosclerotic plaque calcification essentially involves the same biological processes as in normal bone formation 98. Studies have characterized the mineral element of vascular calcification where the calcium deposits primarily exist in the form of HA, again, similar to that seen in bone 99, 100, 101, 102. In addition, the expression of numerous key mediators of bone formation and bone structural proteins are present, such as MGP, OPG and OPN 101, 103, 104, 105, 106. Nonetheless, although the sequence of events leading to normal bone formation is well known, it is still unclear through which specific mechanisms vascular calcification occurs 86, 98. Calcification of atherosclerotic plaque has been attributed both beneficial and deleterious effects 107. Mathematical models predict that micro‐calcifications of the thin fibrous cap of atherosclerotic plaque local stress concentrations that lead to interfacial debonding and plaque rupture 108, 109. And that the effect of micro‐calcification is increased with decreasing cap thickness 110. In agreement with these predictions, histological examinations of ruptured human lesions found that rupture commonly occurs in areas of maximum circumferential stress 111. Rupture in sites of lower stress suggests heterogeneity of plaque constituents, resulting in local stresses 110, 111. However, post mortem *in vitro* imaging of human atherosclerotic lesions found that microcalcification of the fibrous cap was rare 108. Analysis of both stable and ruptured human lesions found that the lipid content of the lesions correlates with maximum circumferential stress and not with calcification 112.

Increased calcium levels promote mineralization and influence various mechanisms in VSMCs that result in increased susceptibility to matrix mineralization 84. Elevated calcium and phosphate induce VSMC calcification *in vitro*, causing VSMC phenotypic change 85, 113, 114. In a calcified environment, VSMC populations contain cells that undergo phenotypic transitions to osteocytic, osteoblastic and chondrocytic cell types 86, 115. This phenotypic change is because of loss of VSMC markers and the gain of osteochondrogenic markers, including alkaline phosphatase (ALP), OPN and RUNX2 86, 116, 117. Moreover, ectopic calcification may be a result of the loss of mineralization inhibitors. These include ectonucleotide pyrophosphatase/phosphodiesterase 1 (ENPP1), ATP‐binding cassette transporter subtype 6 (ABCC6), the CD73 ectonuclease and fibrillin 1 (FBN1) 118.

It has yet to be discerned whether human vascular calcification causes, or is caused by, the expression of bone‐related genes. Therefore, focus on the very early stages of ectopic calcification is critical. Explorations into this may be possible through the use of animal models, where disease progression and the underlying causes can be examined.

A number of popular rodent models exist. Non‐uraemic models include transgenic mice that are deficient in known calcification inhibitors, such as Fetuin‐A 119, MGP 120 and OPG 121. Vascular calcification can also be induced in rats by high doses of warfarin, which induces rapid calcification of vascular elastic lamellae and aortic valves 122. These rats are phenotypically similar to MGP knockout mice, suggesting similarity in underlying mechanisms 122. Uraemic models of vascular calcification include the 5/6 nephrectomy rat. In this animal, renal failure is modelled by total nephrectomy of one kidney and the ligation of 2/3 of the extra‐renal branches of the renal artery of the contralateral kidney 123, 124. These rats develop mild calcification predominantly in the aortic arch 125. This calcification is exacerbated and accelerated by a high phosphate diet 126, or 1,25(OH)2 vitamin D3 treatment 127, 128, 129. Uraemia can also be induced with excessive dietary adenine, of which a metabolite, 2,8 dihydroxyadenine, precipitates in the kidney inducing renal failure 130. When fed a high phosphate diet following a high adenine diet, rats develop severe vascular calcification 131. While several rodent models are presently employed in the field, a robust large animal model of vascular calcification has yet to be established. A brief summary of large animal vascular calcification models utilized to date can be found in Table 2.

**Table 2:** Examples of large animal models of vascular calcification

Animal model	Key findings	Reference
*Pig*	Substantial expression of ectonucleoside triphosphate diphosphohydrolase 1 (eNTPD1, CD39) and ecto‐5′nucleotidase (e5NT, CD73) in aortic valve interstitial cells (VICs) and valve endothelial cells (VECs)—may be important in valves. Both factors influence extracellular nucleotide concentrations, thus may be important in valve pathology.	Kaniewska et al. 2014 19
*Horse*	Sporadic calcification of large arteries (in the tunica media) in racehorses mainly observed in the pulmonary artery (also in aortic, pulmonary and carotid trunks). Observed disorganized collagen and elastin fibres, and presence of chondrocyte/osteoblast‐like cells. Analysis revealed the mineral found was consistent with hydroxyapatite.	Arroyo et al. 2008 141
*Cow*	Bovine aortic smooth muscle cell (BASMC) calcification induced with β‐glycerophosphate. Results found mineral deposition in basal matrix of multilayered areas, presence of extracellular matrix vesicles, calcifying collagen fibrils and calcified nodules. Osteopontin (OPN) inhibited calcification dose‐dependently, but did not inhibit alkaline phosphatase activity or decrease phosphorus levels in culture medium.	Wada et al. 1999 290
	BASMC calcification induced with β‐glycerophosphate or inorganic phosphate. Found increased mRNA expression of decorin (protein expressed in skeletal tissues), and overexpression and supplementation of exogenous decorin with calcification medium increased BASMC mineralization.	Fischer et al. 2004 291
	BASMC calcification induced with inorganic phosphate. Tropoelastin potentially inhibits calcium deposition in the cultured BASMCs through interactions with elastin binding protein (EBP).	Wachi et al. 2004 292

A number of vascular calcification investigations have involved studies of the *ENPP1* gene. Loss‐of‐function mutations in the *ENPP1* gene, which encodes ENPP1, also known as plasma cell membrane glycoprotein 1 (PC‐1), have been associated with rare human genetic disorders 132, 133, 134, 135. Mutations of this gene are linked with a genetic deficiency in pyrophosphate levels causing a life‐threatening disorder known as Generalized Arterial Calcification of Infancy (GACI), a rare autosomal recessive disease characterized by arterial calcification, fibrosis and stenosis, which leads to premature death in neonates 84, 93, 132, 134, 136, 137. As a recessive disorder, the most severe mutations are those that associate with the loss of enzymatic function 93. The significance of *ENPP1* mutations is seen in infants with GACI, who often die with extensive vascular calcification afflicting nearly all arterial beds, including the coronary arteries 93.

The genetically engineered *Enpp1* null mouse model has been used extensively to investigate medial aortic calcification. However, the degree of calcification in this model is significantly milder than in affected patients. Therefore, there is a clear need for a more physiologically comparable large animal model to allow for the development of targeted therapeutic treatments for this devastating rare disease.

Reports of calcification in large arteries of racehorses have been made, including the aorta, pulmonary artery and carotid arteries 138, 139, 140. Arroyo *et al*. (2008) investigated the prevalence, distribution and severity of vascular calcification in both young adult Thoroughbred and Standardbred racehorses. This study showed material consistent with HA, found in vascular calcification, to be present in calcified lesions. Microscopic evaluation revealed thinned, fragmented and calcified elastic fibres in the tunica media of the pulmonary arteries, which were encompassed by dense collagen matrix 141. Furthermore, both breeds and sexes appeared to be similarly affected 141. Studies using these horses may be useful for human calcification research, although it is important to note that calcification can be found in relatively early ages in horses (below 5 years of age), especially in those with a racing background 139. What contribution training and athleticism contribute to these changes is not known, but horses do develop heart valve changes as they progress through training programmes. Ectopic calcification occurring without experimental induction may make the horse a useful model of disease progression, as well as tissue being available from those horses that are euthanized.

At present, there are a limited number of medical approaches with the potential to treat or prevent the progression of vascular calcification and other associated conditions, such as aortic stenosis. An example is the use of the bisphosphonate etidronate, which is currently used for the treatment of GACI 142. Bisphosphonates are strong inhibitors of osteoclast activity, and are widely used in clinical practice to prevent bone loss associated with conditions such as osteoporosis, Paget's disease and metastatic bone disease 143. The prolonged use of etidronate can have undesirable effects, such as severe skeletal toxicity as reported in a 7‐year‐old GACI patient 144. Because of this, the use of etidronate requires close monitoring when administered.

A more recent study describes a potential treatment for vascular calcification using enzyme replacement therapy 145. Daily subcutaneous administration of ENPP1‐Fc, a soluble, recombinant protein with the extracellular domain of ENPP1 fused to the fragment crystallisable (Fc) region of human immunoglobulin G1 (IgG1), was effective against GACI in a mouse model 145. This very promising preclinical study may be the first step to clinical trials with enzyme replacement therapy in GACI patients.

### Aortic dysfunction and FBN1 in Marfan syndrome

Diseases of the aortic root and ascending aorta are frequent causes of aortic regurgitation. Aortic regurgitation can be a consequence of abnormal aortic leaflets, as well as structural defects in the aortic root and annulus 18. Various congenital faults can lead to aortic regurgitation, for example Marfan syndrome (MFS, OMIM 154700), which is an autosomal dominant connective tissue disorder 18, 27. Mutations of the *FBN1* gene are linked with aortic aneurysms and elastic fibre calcification observed in patients with MFS 26, 27, 146. The fibrillin proteins, predominantly FBN1, are the major structural components of the 10‐nm microfibrils of the ECM 147, 148. They are also involved in regulating the bioavailability of TGF‐β. FBN1 is a 350‐kDa glycoprotein, which polymerizes and aggregates to form flexible extracellular microfibrillar structures 27.

Patients with MFS show a broad range of cardiovascular defects. These include thoracic aortic aneurysms that result in aortic dissection, rupture or both 149. Dilatation of the root of the aorta leads to failure of aortic valve occlusion resulting in valve insufficiency. Additionally, MFS can involve dysfunction of the mitral valve (myxomatous thickening, prolapse and regurgitation), and medial degeneration morphologically similar to that observed during ageing, in idiopathic aortic aneurysms, and in sufferers of aortic valve disease and hypertension 27. Prophylactic treatment with beta blockers and angiotensin II receptor antagonists (for example losartan) has proved effective in reducing the rate of aortic dilatation and hence aortic valve dysfunction 150, although there are no treatments for the underlying defect in the FBN1 protein.

Cattle have been established as a model of human MFS. There are a number of spontaneous *FBN1* mutations in cattle, resulting in a condition that shares many of the clinical and pathological manifestations of human MFS 151, 152, 153. Diminished expression of fibrillin has been found in cultured bovine MFS dermal fibroblasts and BASMCs, similar to the findings in human MFS, and pulse‐chase metabolic labelling experiments verified decreased incorporation of fibrillin into the ECM 154. Mutations in *FBN1* have also been detected in cattle afflicted with MFS, with similar clinical features as observed in human MFS 153, 155.

The FBN1 protein is suggested to be a potential inhibitor of vascular calcification, because mice with a knock‐out of *FBN1* displayed ectopic calcification 156, 157. A large animal *in vitro* study with cultured bovine aortic smooth muscle cells found that with accelerated calcification the expression of FBN1 was reduced 158. These findings may suggest pathological interactions between MFS and vascular calcification, adding to the complexity of the calcification process.

A related condition is bicuspid aortic valve (BAV) which is the most common congenital valve abnormality, with an incidence of around 1% 159. This condition is responsible for nearly 50% of surgeries for isolated severe aortic stenosis 160. Although the phenotype is extremely variable, BAV can be familial, with some cases resulting from mutations in *NOTCH1* 38, 161, 162. The bicuspid valve undergoes age‐associated calcification similar to the tricuspid valve, but at a younger age, so that most BAVs have significant calcification by the time the individual reaches 40 years of age 163. In mouse models, BAV has been associated with *Nos3*, *Gata4*, *Gata5*, *Gata6* and *Nkx2.5* mutations 160, 164. Patients with BAV are also at risk of thoracic aneurysms, and abnormalities of FBN1 protein have been demonstrated in the VSMCs of BAV aorta 165.

## Atherosclerosis

Atherosclerosis is a chronic inflammatory disease, and affects medium to large sized arteries 166, 167. It is a major CVD, which can often lead to stroke, myocardial infarctions and peripheral vascular disease in humans 166. Various animal models have been developed and have greatly contributed to the understanding of this disease. Large animal models of atherosclerosis have been generated, including the rabbit, pig, goat and non‐human primates 166, 168.

Initially, atherosclerotic lesions consist of non‐protruding fatty streaks composed of lipid loaded macrophages known as foam cells. As the lesion progresses, VSMCs switch to a proliferative synthetic phenotype, which produces excessive amounts of collagen. As macrophages, foam cells and VSMCs accumulate, the lesion intrudes into the lumen causing disturbed flow. This disturbed flow exacerbates endothelial dysfunction, further provoking the lesion. The increased involvement of VSMCs, along with extracellular accumulation of oxidized LDL and necrotic cell debris, results in structural weakening. Vulnerable lesion rupture results in thrombosis of the vessel and ischemia of the downstream tissue 169, 170.

Atherosclerosis shows a tendency to develop in areas of disturbed or low shear stress, such as bifurcations and curvatures. Indeed, turbulent flow causes endothelial dysfunction *in vitro* 171. Hyperlipidaemia is also an important factor in the development of atherosclerosis. The causal relationship between LDL and atherosclerosis has been extensively studied 169, 170, 172, 173, 174, 175.

The ideal model of atherosclerosis would develop lesions that progress through all stages of the disease, from fatty streak development to unstable plaque rupture. Murine models offer several advantages in general as stated above. For the study of atherosclerosis, the most commonly cited advantages are the short time frame of plaque development, and the comparative ease of genetic manipulation. Wild type mice, however, rarely develop atherosclerosis. Unlike humans, the major circulating lipoprotein in mice is HDL, rather than LDL, which is the key player in atherosclerosis progression in humans. Additionally, mice lack cholesteryl ester transfer protein (CETP) 176, exacerbating the resistance of these animals to atherosclerosis. CEPT facilitates the exchange of cholesterol and triglycerides between HDL and Apo lipoprotein B, simultaneously decreasing HDL while increasing LDL 177. Clinically, low CETP activity is associated with decreased CVD risk 177. Overcoming the atheroresistant phenotype of mice requires genetic manipulation of their lipoprotein metabolism to produce a more proatherogenic phenotype. The two most commonly used models are the Apo lipoprotein E deficient (ApoE^−/−^) mice and the LDL receptor deficient (LDLR^−/−^) mice.

ApoE is produced by the liver and macrophages and is incorporated into circulating lipoproteins. Through binding of LDLR and LDLR related protein ApoE mediates the clearance of LDL and very low density lipoprotein (VLDL) from the circulation 178. Complete deficiency of ApoE in humans is rare. However, the ApoE2 isoform binds LDLR poorly and has a high prevalence in patients with congenital type III hyperlipoproteinemia 179, 180, 181. The genetic ablation of ApoE in mice results in significantly increased circulating VLDL 182, 183. These mice reliably and rapidly develop atherosclerotic lesions; as a result, they have been widely employed in the study of atherosclerosis 182, 183. Despite their usefulness and reliability, the predominant circulating lipoprotein in these mice is VLDL, not LDL, which like wild type mice is markedly different to humans. These animals are also dramatically hyperlipidaemic when fed a chow diet 183. This hyperlipidaemia is greatly exacerbated when fed a high fat diet 183. ApoE has also been demonstrated to possess immuno‐regulatory, anti‐inflammatory and antioxidant properties 184, 185, 186, 187. In light of the role that low chronic inflammation plays in the progression of atherosclerosis, the rapid lesion progression, extreme hyperlipidaemia and pro‐inflammatory state may be considered limitations rather advantages of this murine model.

LDLR^−/−^ mice are more moderate models of atherosclerosis compared to ApoE^−/−^ mice. These mice gradually develop atherosclerotic lesions on a chow diet, which can be accelerated by high fat feeding 166, 183. In a closer approximation to the human disease, the predominant lipoprotein in these mice is LDL 166, 183. LDLR deficiency in humans is the most common cause of familial hypercholesterolemia 188, 189. The LDLR^−/−^ model has been further manipulated to generate mice that express only apoB100 190 or transgenic human apoB100 191. In both cases, these mice develop accelerated atherosclerosis on a standard chow diet 190, 191.

In mice, atherosclerotic plaques predominantly develop in the aortic sinus, aortic arch and brachiocephalic artery, in contrast to human plaque, which primarily develops in the carotid arteries, the coronary arteries and the aortic arch 16, 166. Although significant insight into the initiating mechanisms has been gained from mouse studies, it is noteworthy that the progression, response to treatment and regression of atherosclerotic plaque vary significantly between vascular beds 192, 193. Another important limitation of mouse models is the rarity of advanced coronary lesions that progress to rupture and thrombosis 166, 194. This is a common complication of atherosclerosis in humans that cannot be modelled in these animals.

One ethical and practical concern when designing experiments involving high fat fed mice or ApoE^−/−^ mice is their tendency to develop eruptive skin lesions and ulcerative dermatitis 195, 196. This is a source of significant pain and inflammation, and often requires premature humane euthanasia 195, 196.

Spontaneous atherosclerosis can occur in swine and ruminant species 197, 198, 199, 200. Experimental induction of this disease has been performed in calves and goats, with reported characteristics similar to human atherosclerosis 168, 201, 202. In a study by Hines *et al.* (1985), young male goats were used to assess the effects of dietary calcium and cholecalciferol on plasma and tissue cholesterol concentrations, the distribution of total lipid in the body, and aortic and plasma concentrations of calcium and magnesium 168. Outcomes of this study found that this diet may not affect cholesterol and/or total lipid metabolism. However, effects on deposition of lipid and mineral in arterial walls were noted, and aortic calcification, as well as lipid infiltration and plaque formation, may predispose an individual to atherosclerosis 168. Whilst the goat model may be of limited use because of the relatively short amount of time for atherosclerosis to develop compared to humans, further studies with the goat may contribute to increased understanding of the mechanisms underpinning CVDs.

Pigs have also been used as a model for atherosclerosis 203, 204, 205. Atherosclerosis develops slower in pigs compared to mice. Pigs also develop lesions spontaneously, and in time, these lesions develop in the coronary vasculature 75, 206, 207. Atherosclerosis can be induced in pigs through an atherogenic diet 75, 206, 207. HF/HC fed pigs develop complex atherosclerotic lesions that share many of the pathological features of human lesions, including smooth muscle cells, inflammatory infiltrates, foam cells, fibrous caps, necrotic and apoptotic cells, plaque haemorrhage, calcification and expanded extracellular matrices 208, 209, 210, 211. Anatomically, the distribution of lesions in the pig is similar to humans, and more importantly, these include a propensity for lesions to develop in the coronary circulation 16, 208, 209, 210, 211, 212. One proposed reason for these similarities between man and pig is the similarity of lipoprotein profile between the two species 16, 166. A recent study using young adult male pigs fed on a high fat diet for 20–24 weeks assessed the effects of hypercholesterolaemia, with the aim to examine early stage atherosclerosis 204. Significantly greater intima–media thickness of the abdominal aorta, carotid artery and femoral artery, indicated relatively rapid progression of vessel disease 204. This is consistent with past reports in humans demonstrating that increased thickness of the walls of the abdominal aorta and carotid artery can independently predict atherosclerosis, coronary artery disease, myocardial infarction and stroke 213, 214, 215, 216. Associations of CVD risk factors and events have also been associated with increased intima–media thickness of the femoral artery 217.

Spontaneous familial hypercholesterolemia has also been reported in pigs 218, 219. These pigs have the arg94 residue of their LDLR substituted by a Cys resulting in a missense mutation 220, 221. These pigs have excessive circulating LDL and reduced HDL, and as a result, they develop severe atherosclerosis in the coronary and aortic arteries, even while fed a standard pig diet 218, 219. Transgenic familial hypercholesterolaemia miniature pigs have also been produced in order to evaluate atherosclerosis 222, 223. These minipigs recapitulate several of the features observed in human atherosclerosis 223. An important translational feature of pig models is the possibility of percutaneous coronary intervention using human clinical equipment and stents 224, 225, 226, 227, 228, 229. Furthermore, pig models also develop restenosis 226, 230, a common complication of stent implantation that compromises long‐term outcomes 226. On the whole, the porcine model has great potential in uncovering the more specific details in atherosclerotic progression. The minipig is also advantageous, as it requires lower maintenance costs compared to larger animals.

## Diabetes Mellitus Accelerated Atherosclerosis

Cardiovascular complications are common in diabetes mellitus (DM) patients and include cardiomyopathies and accelerated atherosclerosis. DM patients are usually grouped into two different types based primarily on aetiology. Type 1 DM (T1DM) is characterized by hyperglycaemia because of a deficiency in insulin that is the result of autoimmune destruction of β‐cells in the pancreas. Type 2 DM (T2DM), on the other hand, is characterized by hyperglycaemia because of insulin resistance. T2DM is typically preceded by a hyperinsulinaemic euglycemic period. Glucotoxicity and lipotoxicity in T2DM lead to progressive destruction of β–cells, and ultimately hypoinsulinaemia. Commonly, T2DM patients are also obese with some degree of dyslipidaemia. T2DM accounts for 95% of DM patients and is more commonly associated with CVD than T1DM 169, 170.

Researchers have employed several approaches to develop animal models of DM. These approaches are dependent on the type of DM to be modelled. For T1DM, a common approach is ablation of β‐cells with pharmacological agents, such as Alloxan and Streptozotocin (SZT), where these chemicals are taken up by β‐cells and induce free radical formation leading to cytotoxicity 231, 232, 233, 234. SZT treatment in wild type mice has been shown to induce hyperglycaemia, but with a modest reduction in insulin production 235. When fed a high fat diet, these mice show increased size of fatty streaks 235, 236. SZT treatment in ApoE mice results in hyperglycaemia and insulin deficiency 237, 238. Hypercholesterolaemia in these mice is exacerbated by SZT. This increased cholesterol is primarily VLDL and LDL. Of particular importance is the dramatic increase in atherosclerotic lesion area in the SZT mice compared to non‐treated controls 237, 239, 240. The increase in atherosclerosis in these animals has been attributed to hyperglycaemia and the formation of advanced glycation end products 237. Interestingly, SZT‐treated LDLR mice display no differences in atherosclerosis development compared to untreated LDLR mice 241. Pigs have also been employed in studying the effects of DM on atherosclerosis. Yucatan miniature pigs fed a high fat diet and treated with Alloxan develop hypercholesterolaemia and insulin resistance 242. When compared to high fat fed untreated pigs, the high fat fed Alloxan‐treated group showed significantly increased coronary atherosclerosis 243. Similarly, Alloxan treatment in conjunction with high fat feeding increased atherosclerosis in Sinclair miniature pigs 76.

The models described above have provided important insights into the effects of hyperglycaemia on atherosclerosis. These models do however require some degree of β‐cell reduction. This, depending on the degree of reduction, resembles T1DM more than T2DM. A common method to induce T2DM in animals is a dietary approach. For example, high fat, but not high fructose, fed ApoE^−/−^ mice develop fasting hyperglycaemia and hypoinsulinaemia consistent with T2DM 244. These mice have significantly increased atherosclerosis compared to control diet ApoE^−/−^ mice 244. Similarly, high fat, but not high fructose diet, induces diabetes and increases atherosclerosis in LDLR^−/−^ mice 245.

Mice with impaired leptin signalling lack a sense of satiety, and as a result are characterized by excessive feeding and obesity. These mice readily develop insulin resistance. Similar to high fat fed wild type mice, they do not reliably develop atherosclerosis because of the majority of their circulating cholesterol being HDL. To investigate the effects of DM on atherosclerosis, LDLR^−/−^ mice have been crossed with either leptin deficient mice (Lep^ob/ob^) or leptin receptor deficient mice (lepr^db/db^). These mice are obese with hypercholesterolaemia because of both elevated LDL and VLDL 246, 247. These mice also have extensive spontaneous atherosclerotic lesions 247, 248, 249, 250, 251. The primary difference between the two strains is ApoE^−/−^ lepr^db/db^ have high circulating leptin levels 252. Similarly, double knock out ApoE^−/−^ Lep^ob/ob^ and ApoE^−/−^ lepr^db/db^ are also obese with hypercholesterolaemia and insulin resistance 252, 253, 254. Atherosclerosis is accelerated and exacerbated compared to ApoE^−/−^ only mice 252, 253, 254, 255. Hypercholesterolaemia in these mice is extreme, and concerns have been has raised over the interpretation of results from these animals 256.

Ossabow pigs have a natural propensity for obesity 257. When fed a high fat diet, these pigs develop obesity with decreased glucose tolerance and hyperinsulinaemia 258, 259, 260. High fat feeding also induces hypercholesterolaemia with a predominant increase in LDL cholesterol 259, 260, 261. Neointimal hyperplasia and atherosclerosis are significantly increased in the coronary circulation on these pigs 258, 261. Compared to Yucatan pigs, Ossabow pigs recapitulate more closely the metabolic and cardiovascular phenotype of DM and atherosclerosis 258.

## Abdominal Aortic Aneurysm (AAA)

Abdominal aortic aneurysm (AAA) is the tenth leading cause of death in men above 60, with 6–9% of males over 65 year old being affected, and is becoming more common in women 262. Clinical risk factors for AAA include ageing, gender, hypertension, smoking and a family history of aneurysm disease 262, 263, 264, 265. However, the risk of smaller aneurysm rupture in women is greater than in men 264. AAA involves inflammatory cell infiltration, SMC apoptosis in the aortic wall and ECM degradation 266.

As with many CVDs, the lack of samples from healthy or early stage disease patients hinders research. Accordingly, Riches *et al.* (2013) examined the biology of SMCs from isolated porcine carotid arteries to assess the potential of this model for AAA 263. Porcine arterial SMC samples exposed to combined collagenase/elastase treatment in a bioreactor share phenotypic features with cultured end‐stage AAA human SMC samples 263. The study of SMCs from a large animal source and the use of a bioreactor to maintain an *ex vivo* model would be of value in studies of blood vessel wall components in vascular disease.

## Coronary Heart Disease

Coronary heart disease (CHD) is the single most common cause of cardiovascular‐related deaths in Europe and the USA, accounting for almost 380 000 deaths in 2010 in the USA, and around 74 000 deaths in the UK 4, 267, 268. Various large animal models have been generated through the induction of coronary artery narrowing or occlusion. In a pig model of atherosclerotic CHD the site and time point of coronary occlusions were unpredictable and thus this model is inappropriate for research into CHD‐related heart failure, where it is important to characterize the precise progression of the disease 267. The response to injury in the coronary arteries of pigs is comparable to that in the human 269, 270. As the biological processes seen in arterial repair are similar between the pig and human, these pre‐clinical studies are extremely valuable 6. Other models of heart failure involve surgically constricting the coronary arteries or artificially producing intracoronary embolisms. This approach has been used in dogs and pigs (reviewed in 267).

## Future Directions: Potentials of Large Animal Transgenic Models

There is still much progress to be made in the field of CVDs, and the generation of more suitable large animal models, such as the pig, would be highly valuable in examining the underlying processes that lead to the initiation and progression of this disease. As will be briefly commented on below, the use of novel genetic engineering techniques may play a role in furthering our understanding of CVDs.

## Use of Transgenic Tools

Substantial progress has been made in the fields of genetic manipulation and molecular cloning. These tools create a large window of opportunities for creating refined models for future biomedical research. Such models will facilitate studying the pathophysiology of human diseases, and be instrumental in improving and developing new diagnostic tests and therapeutic approaches.

Transgenic model systems have been established using organisms like the fruit fly (*Drosophila melanogaster*), rodents and zebrafish 271. Although these models are highly informative, they do not sufficiently emulate the complexities of human biology. Consequently, the models of human disease often do not adequately mimic the human condition. There is now increased potential for genetically engineered large animal models of human disease. In particular, the pig is becoming an increasingly relevant model organism for this approach to developing models. This is largely because of closer similarities with humans in terms of anatomy, genetics and physiology than the classical animal models. Pigs are relatively easy to breed, produce large litters, are available in a range of genotypes and phenotypes and provide access for biopsies and post‐mortem samples 271. As mentioned previously, miniature pigs have already proven to be valuable in biomedical research, including the field of cardiovascular biology 222, 223, 272.

**Figure 4:**
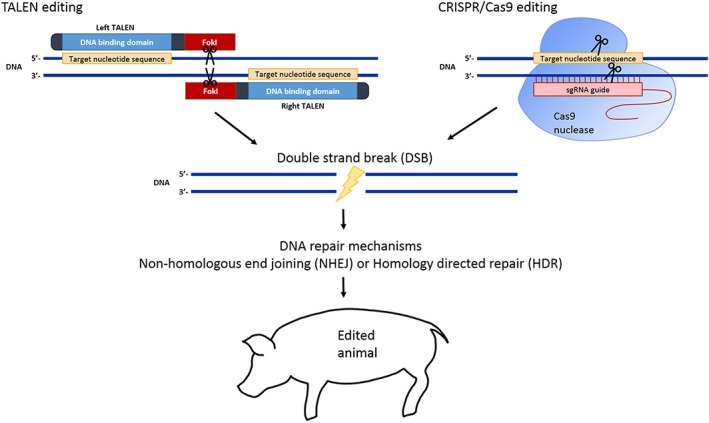
TALEN or CRISPR/Cas9 systems of gene editing. After zygote injection of customized transcription activator‐like effector nuclease (TALEN) mRNA or Cas9 mRNA/single guide RNA (sgRNA), the gene of interest can be targeted and cut to produce a double strand break (DSB). TALENs work in pairs as the FokI endonuclease requires dimerization in order to produce a DSB. Various CRISPR/Cas9 systems exist for different applications. Fundamentally, the sgRNA directs the Cas9 nuclease to the site of targeting. After a DSB is created, DNA repair mechanisms occur via two main pathways: non‐homologous end joining (NHEJ) and homology directed repair (HDR). NHEJ leads to insertion/deletion mutations (indels), whilst HDR can be used to insert desired sequences. Through these mechanisms, an edited animal can be produced

A major goal in biological research is to further our understanding on the relationships between genotype and phenotype. Traditional approaches in understanding gene function have been restricted because of the lack of required tools for gene customization 273. More recently the possibilities of gene customization have expanded rapidly through the development of new and innovative technologies in the field of genome engineering, such as the use of zinc finger nucleases (ZFNs), transcription activator‐like effector nucleases (TALENs) and clustered regularly interspaced short palindromic repeats (CRISPRs). These next generation technologies serve as powerful tools for gene knock‐out and targeted manipulation of genomes (Figure 4).

The first widely used genome editor was the ZFN 274 and along with TALEN technology has been effective in genome editing of cultured cells and numerous species leading to the production of transgenic rats, zebrafish, and pigs 275, 276, 277, 278. More recent to the TALEN editing platform, the CRISPR and Cas9 (the latter of which is a class of RNA‐guided endonuclease) system is rapidly evolving as a novel genome editing technology 279. This is due its ability to achieve precise genome editing to induce the specific mutations that have been observed in human patients, in a manner that leaves no molecular footprint within the target genome.

The CRISPR/Cas9 technology has also been used in large animals. Gene knockout in goats has achieved an efficiency of 9–70% of induced mutations in primary fibroblasts, with success in generating cloned goats with bi‐allelic mutations, although cloning efficiency was low (1.1%), similar to other groups 280, 281. CRISPR/Cas9 has also been used in pigs, where the von Willebrand factor (*vWF*) gene, whose deficiency causes severe von Willebrand disease in humans, was targeted 282. In this study, 68% piglets born through zygote injection had edited genomes, with 55% of these bearing bi‐allelic mutations and 45% with mono‐allelic mutations 282. The overall high birth and survival rates indicate little toxicity from injecting with Cas9 mRNA and single guide RNA (sgRNA) 282. These studies demonstrate that this transgenic tool can be used in livestock species, and can contribute to numerous applications of large animals in biomedical research.

There are limitations to the use of transgenic models, because of the present lack of information on the molecular biology of large mammalian organisms. Recent efforts have been dedicated to characterising these animals at the molecular level, for example in terms of their genomics, transcriptomics and proteomics. The pig genome has been extensively sequenced 283 as has the sheep genome 284. Information is also available for the genomes of dog, cat, bovine, horse, guinea pig and rabbit (see http://www.ensembl.org), all of which can provide natural or transgenic models for human CVD. Although the gene annotation and assembly of the porcine genome are incomplete, genomic comparisons between the pig and human do demonstrate more structural resemblance than between mice and human 285, 286. Transcriptomic analysis of pig RNA also shows greater similarity with human than does mouse 287. With the combination of innovative and efficient transgenic tools coupled with biologically relevant models of human diseases, there is little doubt that major advances in the cardiovascular field will be made in the areas of drug discovery, and targeted therapies for CVDs.

## Conclusions

Large animal models of human disease are valuable resources for identifying and gaining knowledge on the underlying factors in the progression of CVDs and the mechanisms of action. Understanding the critical molecular processes and the role of fundamental drivers that lead to CVDs is important in ensuring successful outcomes of interventional and therapeutic approaches. With the advancements of state‐of‐the‐art genome editing technologies like TALENs and CRISPRs, customisable models can be developed, which will greatly enhance the field of cardiovascular research. This can allow for more translational research potentially leading to treatments for human cardiovascular disorders, both congenital and acquired.

## Conflict of Interest

The authors have declared that there is no conflict of interest.
